# The Hospital. Nursing Section

**Published:** 1903-07-04

**Authors:** 


④ The Hospital.

**Nursing Section.**

Contributions for this Section of "The Hospital" should be addressed to the Editor, "The Hospital" Nursing Section, 28 & 29 Southampton Street, Strand, London, W.C.

No. 875.—Vol. XXXIV. *SATURDAY, JULY 4, 1903.*

### Notes on News from the Nursing World.

### QUEEN ALEXANDRA'S NURSES.

At the annual meeting of the Soldiers and Sailors' Families Association last week it was stated, in reference to the nursing branch, that 20 nurses are now employed at a cost of £2,064 14s. 4d. to the Association. The total number of visits paid by these in the year was 55,631, and the number of cases nursed was 4,576. It is satisfactory to learn that this branch is expanding year by year, in illustration of which it was mentioned by Sir James Gildea that there are now six of Queen Alexandra's nurses fully employed in Malta, most of their patients being among the families of sailors.

### IN AID OF THE HOME FOR LADIES OF LIMITED MEANS.

On Friday, July 3, a special matinee will be given at the Garrick Theatre, with the Queen as patroness, in aid of the Hospital for Ladies of Limited Means, in Harley Street. This hospital was founded in 1850 by the Viscountess Canning, and organised by Miss Florence Nightingale for the purpose of providing, at a very moderate cost, a Home for the medical and surgical treatment of ladies unable to afford the expense of doctors or nurses in their own abodes. It is specially intended for the wives, daughters, and sisters of the clergy, naval, military, and professional men, and for governesses, artists, and other ladies of limited means who are admitted with the approval of the committee. The medical and surgical aid is given gratuitously, and the Home has now, for more than half a century, been one of the most deserving and useful institutions in the Metropolis quite fulfilling the expectations of its illustrious founders. The programme at the matinee will consist of "The Squire," by Mr. A. W. Pinero, which is to be revived for the occasion, with Miss Kate Rorke as the heroine, and "Gringoire." Prices will be as usual, and tickets can be obtained from the hon. secretary, Miss Violet Daubeny, Artillery Mansions, 75 Victoria Street, S.W.

### PRINCESS LOUISE AND SOUTH LONDON NURSES.

On Tuesday afternoon Princess Louise presided at the meeting of the South London District Nursing Association, in Battersea Town Hall. The Princess, who was accompanied by the Duke of Argyll, drove with an escort of the Surrey Imperial Yeomanry, to Lavender Hill, which was decorated with flags and lined with hundreds of spectators. Her Royal Highness was received by Canon Erskine Clarke, chairman, and other officers of the Association. A bouquet of carnations, stephanotis, and asparagus fern having been offered by Miss Joan Dickinson, the little daughter of the hon. secretary, the Princess, who was wearing a dress of silver-blue silk and a black toque with pink roses, took her place on the platform. Canon Clarke moved a resolution expressing high appreciation of the Association's work, and pledging the meeting to assist in raising funds to carry on and extend its operations, which was seconded by Sir William Broadbent, Bart., and supported by Sir John Furley, C.B., who said that it was the duty of everybody to see that a high standard of nursing was maintained, and not to allow it to degenerate into a spasmodic or ephemeral thing. All the speakers referred in terms of the highest praise to the work of Miss Bullock and the nurses among the poor and in the schools. The resolution was carried unanimously. About eighty little boys and girls, representing the parishes and schools of the district, as well as private persons, then presented purses containing in all more than £280. The Nurses' Purse, containing £10 15s., was offered to the Princess by a little patient, Grace Holloway. The Duke of Argyll returned thanks on behalf of the Princess, and wished the Association every success. It was evident, he observed, that its work was appreciated by the entire borough, and he was sure that they would not fail to support it as it deserved. Before leaving, Princess Louise graciously signified her willingness to become President of the Association. The collection made by the nurses at the close of the meeting amounted to about £30.

### MEETING OF THE CENTRAL MIDWIVES BOARD.

A meeting of the Central Midwives Board was held at the Privy Council Office, Whitehall, on Thursday, last week, Dr. Champneys in the chair. A letter was read from Dr. Kaye, County Medical Officer for the West Riding, on behalf of a conference of county medical officers, lately held, suggesting that the Board should receive a deputation from the conference to hear the suggestions of the latter with regard to the administration of the Act in county areas. The proposal was favourably received by the Board, and the secretary was instructed to arrange a meeting in London at an early date. The Chairman reported on the question of office accommodation and further instructions were given to the secretary on the subject. The draft form of the midwives' roll as submitted by the secretary was considered, approved, and adopted. Alternative draft forms of the certificate required by the Act having been submitted by the secretary, the Board decided that the certificate should in each case show on the face of it the qualification in respect of which it was granted. The appropriate forms were approved and adopted.

### THE NURSING ARRANGEMENTS AT NEWCASTLE ROYAL INFIRMARY.

It cannot be affirmed that the fine new infirmary, which is being erected at Newcastle-on-Tyne, is an extravagance. Apart from the vital point that the old institution has long since ceased to be sufficient in size, or in any other respect, to the requirements of the patients, the conditions under which the nurses perform their duties are so bad that we are surprised the authorities have been able to keep the staff together. In an interview with our Commissioner, which appears in another part of the paper, the matron intimated that it is necessary to retain the probationers as staff nurses or sisters, after they have served their three years of training, if only because very few nurses trained elsewhere would put up with the heavy work in the wards and the discomforts outside. Both these are due to lack of room in the building. The quarters of the minority of nurses who sleep in it are described by the matron as "shockingly inadequate," and when it is added that they sleep two or three in a room, the accuracy of the description will not be doubted. But even worse than this is the makeshift arrangement by which the majority of nurses sleep in different parts of the city, involving a walk in the early morning, and late in the evening, of 10 or 15 minutes. The matron's defence is, that no other arrangement was possible in existing circumstances, but it is strange that in such a go-ahead city as Newcastle it has been tolerated so long. It is true that a better time is coming when the nurses of the Royal Infirmary will have a suitable home of their own, but this does not alter the fact that so many of them have now for some years been exposed to all the serious disadvantages of sleeping away from the hospital, without the protection they have a right to expect. We hope too, that when the home is finished, an effort will be made to supply the nurses with some kind of library. At present neither books, magazines, nor newspapers are provided by the Committee.

### WEST HAM HOSPITAL.

An "At Home" with a special object was given in splendid weather on Saturday last at the West Ham and East London Hospital, Stratford. The proceedings began at half-past three, when a very large number of friends and well-wishers were received by the matron, the chairman of the hospital, and his wife. In the entrance hall a band was stationed, made up of five nurses of the establishment—two at the piano, two mandolinists, and a fifth who performed on the violin. After tea there were two entertainments, one in the Dispensary Hall and another in the Drill Hall of the High School. Both were admirable, and there was no charge for admission, the price being optional! The money was received in tambourines held by nurses. Subsequently, the extension scheme was explained by the Bishop of Barking, Dr. P. J. S. Nicoll, and the Mayor of West Ham. The West Ham Hospital is bounded on one side by a road, on the other by a Girls' High School. The latter, with a large hall, class-rooms, and an extensive playground, is for sale and the committee of the hospital are most anxious, if possible, to secure this, the only possible "lung," for the good of the patients. They hoped that if visitors saw exactly how matters stood for themselves, babies sleeping "heads and tails" and little children upon mattresses upon the floor owing to the crowded space, they might help to raise the £3,000 which is now all that is wanted to make up the total of £15,000 required. Letters of regret for absence were read from the Duchess of Marlborough and the Countess of Warwick.

### THE LONDON COUNTY COUNCIL AND THE MEDAL QUESTION.

It was recently stated that the London County Council had issued an order forbidding nurses and attendants engaged at the institutions under their jurisdiction to receive medals. The order, it was affirmed, was made in consequence of the decision of the Society for the Protection of Life from Fire to present medals to the nurses and attendants who were conspicuous in their attempts to rescue patients at the Colney Hatch fire. As a matter of fact, however, no order has been issued by the London County Council. In reply to a letter from the secretary of the Society for the Protection of Life from Fire, offering to submit to his trustees for consideration the names of such of the staff at Colney Hatch Asylum as particularly distinguished themselves in rescue work on the occasion of the fire, the sub-committee of the asylum declined the proposal with thanks on the ground that they considered it most difficult, if not impossible, to differentiate and single out those of the staff who were deserving of special reward. To this decision they were helped by the action of the Asylum Workers' Association, which, as our readers know, has presented to the asylum at Colney Hatch a brass tablet recording the "heroic conduct and self-sacrificing devotion to duty displayed by members of the staff." We do not think that in these circumstances the censure passed upon the London County Council in some ill-informed quarters is at all deserved. There is nothing in their treatment of the offer to justify the assertion that they are the slaves of red-tape policy, or are even opposed to the acceptance of medals by their nurses when there is no practical difficulty in awarding them.

### THE PASSING OF THE UNTRAINED MATRON.

The remarks of Mr. Baldwyn Fleming, who, as Local Government Board inspector, attended the meeting of the Christchurch Guardians in reference to the proposed alteration of the nursing arrangements at the workhouse infirmary, may be commended to the attention of Boards of Guardians in general. Mr. Fleming, having been told that it was in contemplation to make the matron head of the nursing staff of four charge nurses and four assistant nurses, promptly rejoined that that was out of the question. It would, he said, be quite impossible for the matron, who was not a nurse, to be the head of the nursing staff; and he declared that, under the rules of the Local Government Board, it was necessary in workhouse infirmaries where there were more than two nurses that there should be a superintendent nurse. In fact, he did not see how the Guardians could possibly work their nursing staff without a superintendent nurse at the head, and he gave point to this statement by the observation that no person could be the head of the nursing staff who was not competent to direct the nurses in any emergency, and she could not do that unless she was a fully-trained nurse. A Guardian then suggested that one of the four head nurses whom it was proposed to appoint should be superintendent nurse. But Mr. Fleming demurred to the reduction of the staff thus outlined, and also advised the Guardians to consider whether £40 a year was a sufficient salary for the occupant of such an important post as superintendent nurse in their infirmary. Finally, it was asked whether if the board would decide to appoint a resident medical officer, a superintendent nurse could be dispensed with; and Mr. Fleming replied that such an appointment would rather emphasise than do away with the necessity of electing a superintendent nurse. The result of the inspector's firm attitude was that the Christchurch Guardians decided to fall in with his views, and will now have an adequate nursing staff with a qualified superintendent at its head. It is clear, also, that the Local Government Board intend that this shall be the case everywhere, for Mr. Fleming told the Christchurch Guardians that this Board have not sanctioned any arrangement by which nurses are controlled by a person who is not a fully qualified trained nurse.

### AN UNTENABLE POSITION.

In the Chancery Division last week Mr. Justice Farwell had before him an action by the executors of the late Colonel Des Barres, who applied to the Court to set aside a transfer of stock valued at £1,450, on the ground that the deceased was induced to execute the deed of transfer to Miss Irene Kate Hayles, a professional nurse, by means of undue influence on her part. It was also sought to recover a quantity of jewellery and plate, said to have been taken away by Miss Hayles, but which she contended were gifts on his part. She also denied that she exercised undue influence on him. These issues were not argued, because at the close of the case for the plaintiff it was announced that the parties had arrived at a settlement, the terms of which were not stated. But Mrs. Boake, the proprietress of a Nurses' Home at Westbourne Grove, deposed that Miss Hayles was sent by her to attend Colonel Des Barres, and that on being informed by the nurse that her patient was making her a present, witness told her that her position was untenable, and requested her to return to the Home. She added that Miss Hayles refused to do this, and severed her connection with the Home. We entirely agree with the view taken by Mrs. Boake. Any nurse who receives presents from a man she is nursing renders her position untenable, and is herself solely to blame for any unpleasantness that may follow.

### DEPORTMENT IN AMERICA.

It is a custom with some people to institute comparisons between English and American nurses for the purpose of showing that the advantages are all on the side of the latter. We commend to such persons a letter from the head nurse of a hospital in the United States to an American journal of nursing, in which she inquires whether surgical work has a tendency to make both surgeon and nurse coarse and brutal in thought and action. The answer is obviously that no member of either profession, whether male or female, who has a spark of proper feeling can be made coarse or brutal in the discharge of duties. But the reason given for this inquiry suggests a deplorable state of affairs in some institutions on the other side of the Atlantic. Describing a scene witnessed by herself, the nurse says that at the operation in question there were three physicians and the same number of graduate nurses present, and that "a continual conversation was maintained in which oaths, low jests, and unkind remarks about the unconscious patient were most prominent." Other details are given, but this is the essence, and it is certainly enough to justify apprehensions lest the case be other than an isolated instance. There may be room for improvement in the department of individual English nurses, but we cannot conceive them being placed in the position of being compelled to listen to oaths, low jests, and unkind remarks in the operating theatre, or of degrading themselves by laughing, as these American nurses are said to have laughed, at the profanity and rude personalities of the operator.

### A POPULAR MASTER AND MATRON.

The completion of ten years of service by Mr. and Mrs. Hull, the master and matron of the Union Workhouse at Stockport, was marked last week by the presentation to them by the staff of a case of silver dessert knives and forks in token of esteem and respect. The presentation was made by the medical officer, Dr. Bale, who contrasted the state of things when the master and matron were appointed with that of the present day. Then, the entire staff numbered twenty, three only being nurses, and of the nursing arrangements the less said the better. Now, there are sixty-one officers, of whom thirty are nurses and attendants, nine being sisters and staff nurses, and there are three nurses' homes, with every comfort. Dr. Bale said he was told that the Local Government Board Inspector, on the occasion of his last visit, said that although Stockport Workhouse was the worst structural building in his district it was the best administered, and the improvements, he added, were largely due to the energetic and combined efforts of the master and matron.

### TONBRIDGE UNION INFIRMARY.

Four probationers, who have nearly finished their three years' training at the Tonbridge Union Infirmary, have been successful in passing the final examination. The Board of Guardians have expressed their appreciation of the work of the superintendent nurse, Miss Tisdell, as to the completeness of the training given to the nurses.

### THE AUTHORITY OF THE MATRON AT POPLAR

The managers of the Poplar and Stepney Sick Asylum have been warned by Mr. Walsh, the assistant Local Government Inspector, that their decision to appoint probationer nurses themselves not only destroys the authority of the matron, but is also contrary to Article 6 of the Board's Order. Yet because the inspector did not call at the Clerk's Office when he visited the asylum, the managers have declined to send any reply to the official communication of the Local Government Board. We trust that, whatever argument may be urged in favour of the course proposed by the managers, the Local Government Board will insist upon the authority of the matron being upheld.

### SHORT ITEMS.

The Queen has been pleased to nominate Nursing Sister Lees to her vacant "Alexandra" bed in the British Home and Hospital for Incurables.—The ss. *Dilwara*, which arrived at Southampton from South Africa on Saturday, had on board Nursing Sisters H. L. Neale and F. M. Hall, who are on leave for 15 weeks.

### The Nursing Outlook.

"From magnanimity, all fear above;
From nobler recompense, above applause,
Which owes to man's short outlook all its charm."

### THE SOURCES OF SUPPLY.

We propose to devote a series of five articles to a careful consideration of the position and prospects of trained nurses in the United Kingdom, in order to try and discover what can be done to remedy the chaotic and disorganised state of the nursing profession. And naturally the first subject which calls for comment is the source from whence the present supply of nurses is being drawn.

Before the advent of Florence Nightingale the hospital nurse belonged to the charwoman class, except in those cases in which religious sisters undertook the work. After the Crimean war a body of devoted women of the educated middle-class trained as nurses; but the main supply of workers was drawn from the upper class of domestic servants. Then about twenty years ago the system of paying probationers who could enter for a short period of training was inaugurated, and was praised and made popular by the late Sir Walter Besant and other writers whose stories reached all classes, and we had a rush of sentimental damsels into the wards, that for a time was half a subject for laughter and half for admiration. For those actuated by mere sentiment were soon weeded out, and those whose characters were more sound aud steady proved excellent material for the making of non-sectarian nursing sisters—capable not only of nursing the sick, but of teaching and training those whose early education had not equalled theirs, and capable also of organising and managing an institution and a business.

The daughters of lords temporal and spiritual did not scorn to serve their three months in hospital, but it was soon found that the higher the social position of a probationer the shorter her stay as a rule. Most of these women did not intend to take up nursing as a life-long profession, nor did their social and family duties allow of it. Nor was it to be desired that women of wealth should take the well-paid positions of a profession chiefly filled by those who depended on their labours for their daily bread. But because the lady of title in an estimable desire to share in the service of the suffering did her small quota of ward work, in snobbish circles the profession of nursing was looked up to as more "genteel" than serving behind a counter, or clerking, or teaching, and there was a rush on the hospitals by that body of women who are the most unsatisfactory of their sex — the poverty-stricken "ladies" with all the *faiblesse* of good birth, too proud to have availed themselves of a public education, too proud to have done practical or manual work, and their whole outlook on life narrowed by having both the disadvantages of poverty in themselves and of wealth in their forebears. And with them went others from the shops and from domestic service who bought medical books second-hand, and always insisted that their fathers were medical men or clergy. They were only partly to blame for these pretensions, for meanwhile several hospitals had made the invidious rule that they would only train "ladies" as nurses.

It is of course to be desired that there should be two examinations of every woman wishing to train as a nurse; first as to her physical capability and secondly as to her mental capability. A nurse must be equally able to lift a patient and to read a prescription. There should also be references as to moral character, but beyond that we believe the hospital world should be a democracy. For the present state of affairs is debarring the most satisfactory material from which the private nurse is made from securing her training. The genteel snob is an intolerable nuisance in a gentleman's house; she is neither fish nor flesh nor good red herring. She has to assert herself constantly with the servants and she does not know how to behave with the family—though she is generally to be found surreptitiously studying a book of etiquette when she ought to be attending to the patient.

And the real lady naturally prefers not to take up private nursing where her position in a house where she might previously have been visitor would be difficult and embarassing. The lady stays in hospital, or on the district, or joins the army or navy. And meanwhile a lady of rank writes to us to say that she finds it impossible to get any big hospital to take for training one of her upper servants who has shown special enthusiasm and ability for the work, and who is strong, sensible, and thoroughly well grounded in elementary education. If we cut off this source of supply of private nurses, if we refuse entrance to the children's nurse, or upper domestic servant, we are wronging a most worthy and hardworking class, and we are increasing the growing irritation of the public against the "genteel" nurse whose object is so often matrimonial and whose advent in a family is too frequently productive of a lawsuit.

Ability, honesty and sympathy are needed in a nurse, but the source of supply ought not to be limited by any social considerations whatever. There is room for all. But the good nurse of high attainments and skill, who regards her work for the sick as a privilege most precious and elevating, must ever command the respect and appreciation of all who have the fortune to come in contact with her. The supply of this type of nurse is always less than the demand. Indeed, were it not for the hope of securing the services of such a woman many doctors and most heads of households would hesitate to employ nurses in private cases. Character, after all, is the true test of value in a nurse, as in a man, and we look forward to the day when the general acceptance of this view will rid the nursing profession of all women who fail to possess this essential qualification. Without character a nurse is not only not indispensable, but may in practice prove to be a danger rather than a help to her patients.

### Lectures on Medical and Surgical Nursing.

By JOHN HOPKINS, F.R.C.S., Medical Superintendent of the Central London Sick Asylum District Asylums, Cleveland Street, W., and Hendon, N.W.

### LECTURE XI.—DISEASES OF THE CIRCULATORY SYSTEM.

THE heart is subject to functional disorders, such as palpitation and irregularity. These are caused by nervous disturbance, and by indigestion, and are of trifling concern. Organic disease may give rise to the gravest state, but often a little care will prolong life for many years, and the subjects of it may live to a good old age. Senile changes are common in people of advanced years especially of the aortic valves. The sack in which the heart lies, the pericardium, may be acutely inflamed. In *pericarditis* the two surfaces of the sack may be glued together by organisable lymph, or there may be such an abundant effusion of serum as to press upon the heart and seriously interfere with its action. In such a case there is danger of the heart suddenly giving out and on no account must the patient be allowed to sit up, lest he die suddenly of syncope.

*Endocarditis.*—Acute inflammation of the lining membrane of the heart is most frequently met with in acute rheumatism. It is often associated with pericarditis. The valves of the left side are those affected. This condition is recognised by hearing murmurs in the heart. After the inflammation has subsided the murmurs generally remain, and indicate permanent damage to the valves, which allows the blood to flow backwards in part. This is known as regurgitation.

*Valvular Disease.*—Mitral regurgitation produces an overgrowth of the muscular substance of the heart. The reason of this is that the blood that has regurgitated has to be pumped over again, and thus the heart has more work to do and grows larger and stronger in doing it. This *hypertrohpy* of the heart compensates for the defective valves. The regurgitation into the auricle causes an engorgement of the pulmonary veins, which increases any tendency to bronchial catarrh there may be. The blood in the right heart is held up too, and the whole venous system tends to be over full. This may give rise to an escape of the fluid part of the blood from the veins into the connective tissues which swell up and the patient is then said to have *dropsy*, and the swollen part is affected with *œdema.* Dropsy only comes on when compensatory hypertrophy fails to force on the blood sufficiently rapidly.

*Mitral Obstruction* is a much rarer condition than regurgitation. Sometimes it occurs in young children. It leads to hypertrophy of the left auricle and atrophy of the ventricle. The auricle overgrows because it has to force the blood through a narrow orifice and dilates, too, because it fails to empty itself. The ventricle grows smaller than natural, having a smaller quantity of blood to throw into the aorta at each beat. In order to supply a sufficient quantity of blood it must beat faster. This condition gives rise to dyspnœa and palpitation. The engorgement of the pulmonary veins gives rise to hæmoptysis.

*Aortic Regurgitation.*—With each beat of the left ventricle blood is thrown into the elastic aorta which expands under the force of the blood-flow, and contracting again, it forces the blood onwards in a steady stream at the same time closing the aortic valves. If these allow regurgitation to take place into the ventricle, this cavity has two currents of blood flowing into it at the same time, and thus being overfilled it dilates. It also hypertrophies, for it has to throw a larger quantity of blood into the aorta at each beat and to stem the current flowing back into it. The effect of this regurgitation on the smallest arteries is to fill them badly and keep them abnormally contracted, so that the patient looks pale through lack of bright arterial blood in the vessels in and beneath the skin. Old people are often the subjects of aortic regurgitation, the valves having undergone calcareous degeneration, lime salts having been deposited in them and hardened them so that they cannot fall together. The special symptom in this condition is attacks of syncope. The patient often turns pale and feels giddy. Sometimes death takes place in an attack, even when the patient is in bed, and so suddenly and quietly may this happen that there is no suspicion of the occurrence until an attempt is made to awake the patient.

*Ulcerative Endocarditis.*—In this condition there is a deposit of fibrin upon the valves, which is due to the presence of bacteria. There is irregular fever and the fibrinous deposit being detached and set free in the blood current is carried into distant parts. These detached pieces are called *emboli.* An embolus carrying with it the bacteria sets up inflammation in the organ in which it lodges round about it, and gives rise to symptoms of disease in that organ.

*Neuralgia* of the heart is frequently observed in diseases of the heart. Patients suffering from heart strain not unfrequently complain of precordial pain. Of much rarer occurrence is *angina pectoris,* which is generally due to calcareous deposit in the lining of the coronary arteries. The calibre of the vessels becomes greatly narrowed, so that the supply of blood to the heart's substance is deficient. In this disease the patient is suddenly seized with a violent spasmodic pain in the heart which lasts for some moments, causes the patient to hold himself in a fixed attitude, wearing a look of great distress, and appearing as though about to die, and he generally does so within three years of the first onset in one of these attacks. Relief is sometimes given by the inhalation of nitrite of amyl, which should be promptly administered. In dealing with cases of heart disease, where there is a threatened failure of compensation, patients are often restored to fairly good health and are able to resume their occupations. Given a case complicated with bronchitis, the dropsy may be got rid of by curing the bronchitis. The heart itself may be acted up, and made to beat with greater power. Drugs are capable of effecting this. When the patient is equal to taking exercise, this is so regulated as to cause the heart to grow and become capable of once more compensating for the regurgitation.

*Disease of the blood-vessels* is general in advanced age and in kidney disease. The coats of the vessels are unable to resist the pressure of blood, and hæmorrhage into the tissues, especially into the brain, is the result. Patients with kidney disease are liable to death from apoplexy. When a large vessel yields before the blood pressure it generally dilates; or at one spot the blood pushes out a little pocket on one side of the artery, which grows steadily larger, pushing everything before it and in the case of bone absorbing it, until at last it bursts either externally or into a cavity, and so causes the death of the patient. The cure of aneurism is effected by causing the blood to clot in it. Rest, drugs, constriction of the artery supplying the blood, arrest of flow by constricting or compressing the artery leading away from the aneurism are all for the purpose of causing the blood to clot. The patient must be kept quiet during treatment, lest the clotting be prevented. Diseased arteries may cause the blood to clot within them. This clot is called a thrombus. Thrombosis of a cerebral artery causes paralysis, and in an artery of a limb it may cause sloughing. In old people it is the cause of dry gangrene. The commonest disease of the veins is a general dilatation of them, mostly seen in the legs, and is due to long standing and constriction of the veins so that the blood cannot flow freely back to the heart. Tight garters, tight stays, and the like are the usual cause. This *varicosity of the veins* gives rise to a series of trouble. It causes a dull aching pain in the limbs, and leads to atrophy of the skin lying over the veins which burst externally. The veins inflame and cause the blood to clot in them. This thrombosis of the veins calls for absolute rest, lest the thrombus become detached and lodged in the lungs. Ulcers, chronic and difficult to heal, are another of the troubles that they give rise to. Relief is obtained by giving external support by means of a carefully adjusted bandage or by an elastic stocking, but care must be taken not to constrict the limb when applying a bandage or the condition will be aggravated.

### The Nurses of the Royal Infirmary, Newcastle-on-Tyne.

A CHAT WITH THE MATRON. BY OUR COMMISSIONER.

The squalid—and only—entrance to the Royal Infirmary at Newcastle-on-Tyne does not give a favourable impression of the venerable institution for the sick and lame poor of the counties of Newcastle-upon-Tyne, Northumberland, and Durham; but there is, at any rate, one excellent reason why no attempt should now be made to render it more inviting. As the House Governor, Mr. Orde, with whom I had a short conversation on the occasion of my visit the other day, hastened to observe, thanks to the bequest of the late Mr. John Hall, the energy of Sir Riley Lord, and the generosity of Mr. Watson Armstrong, the capital of Tyneside should possess in less than two years a hospital worthy of its wealth, its enterprise, and its reputation. The wards are behind the times, and the "operation theatre" is practically only an ordinary room used for the purpose. But the former are admirably kept, and are light and airy; while in none of the modern palatial buildings, where the surgeons have every possible supplementary aids to their skill, are critical operations more successfully performed.

Naturally, when presently I saw the matron, I asked her about the accommodation for the nursing staff, and Miss Aston candidly informed me that it was very bad.

"For that reason," she continued, "the committee hope that the nurses' home which will be attached to the new Royal Victoria Infirmary, and be connected with it by a conservatory, will be completed before the end of the present year.

"Before the hospital itself is finished?"

"Yes, for urgently as better accommodation is required for the patients, the case is still more pressing for the nurses. We can only put up a few of them in the present building."

### Nurses Sleep Out.

"Then," I inquired, bearing in mind the fact that the infirmary stands in close proximity to the railway station, and quite away from homes used for residential purposes, "where do you put up the majority?"

"They sleep in different parts of the city. The night superintendent and 13 night nurses live at Rye Hill."

"How far is that away?"

"It is a walk of from 10 to 15 minutes from here. Twenty-nine more of the nurses occupy three old-fashioned houses in Ravensworth Terrace, about 10 minutes' walk from the hospital, and five probationers are accommodated at the Young Women's Christian Association, a distance of about a mile."

"Then, most of the nurses have to walk a long distance before they enter the wards early in the morning, and again at night when they leave them before they can commence their rest?"

"Yes, unfortunately that is so. I have nothing to say in defence of the arrangement, except that it is the best that could be made under existing conditions. The necessity of living so far away increases the work tenfold, causes a good deal of anxiety, and is altogether to be deplored."

"How do you manage in respect to meals?"

"All the day nurses have their meals in the hospital, but the night nurses have their meals at the Home in Rye Hill. The quarters for those who live here are shockingly inadequate. The sisters have separate rooms, but the staff nurses sleep two or three in a room."

### The Staff to be Increased.

"The new home will be in striking contrast?"

"It will indeed. There will be accommodation for a hundred or a hundred and twenty nurses."

"But you have nothing like that number now?"

"No, there are only 68. We want more, but we cannot have them while we are in this building. Moreover, there will be 400 beds in the new infirmary, about 120 more than we have at present."

"Has the nursing staff been augmented in recent years?"

"Since I came it has been increased from 55 to 68. I have been matron and superintendent of nurses since November, 1895."

"You must have been painfully struck with the deficiencies, especially so far as the nurses are concerned?"

"I had not in my varied experience seen nurses of a great institution so badly provided for. But even during nine years the work has grown very much."

"Has your experience extended to other countries?"

"To several. I was trained at the Nightingale School, aud remained at St. Thomas's for six years. From St. Thomas's I went to the Royal Infirmary at Liverpool; then I had three years' experience as matron in one of the fever hospitals under the Metropolitan Board of Works. I have taken charge of the Government Civil Hospital in Ceylon for two years, was matron of the Colonial Hospital at Gibralter for the same period, and for a year and eight months I nursed and superintended the nursing of yellow fever cases in South America. On my return to England, after a few months' rest, I took up this appointment."

### The Work in the Wards.

"The training here is for three years, I believe?"

"Yes, the regular training; but probationers sign an agreement for two years. It is a convenient arrangement, and when I came here I saw no reason to ask the committee to alter it Of course, we give a modified certificate for the two years, and we only appoint probationers who have been trained for three years as our staff nurses or sisters. If the nurse does well, and is desirous of staying on for another year, which is usually the case, she is permitted to do so."

"How many sisters are there?"

"Nine; there is also an assistant matron and a night superintendent. The wards are staffed according to size; the larger wards have each a certificated staff nurse attached to them. I should like to have every ward provided for in the same way. Among the larger wards are the

### THE NURSES OF THE ROYAL INFIRMARY, NEWCASTLE-ON-TYNE—*Continued.*

two male surgical, with 50 and 48 beds respectively. The two male medical wards have 28 and 27 beds respectively. There is a small mixed medical and surgical ward with 14 beds, a children's ward with 32, a female medical ward with 29, and a female surgical ward where all the surgeons have cases, with 31. In addition there are two small wards where somewhat important surgical cases are treated, and two for female medical cases. We recently, with great difficulty, installed the Finsen light for the treatment of lupus in the basement, and we have also the *x*-ray department. Two nurses are detailed for this work, but they will not supply all the needs if patients increase in number."

### Hours and Salaries.

"Are the hours exceptionally long?"

"Rather, but long passes from two to ten, and four hours off duty alternate days or later if required for special purposes, are given. The sisters enter the wards at 8, and the staff nurses and probationers at 7; they go off at 9 to supper. It would be possible to shorten the hours, but that would involve doing away with the four-hour passes on alternate days, and the nurses prefer the existing arrangement. Nurses who are on duty on Sunday are allowed two hours off. The staff nurses and probationers have three weeks' holiday and the sisters a month."

"Are there any regulations of special interest?"

"The age considered desirable for admission is from 22 to 30. Probationers have three months' trial before they are required to bind themselves for the full term."

"Do they receive a salary the first year?"

"No. The second year they are paid £10, and the third £20. Staff nurses receive from £24 to £30, and sisters from £24 to £36."

### Theoretical Training.

"As to the training," continued the matron, "it is more of a practical than of a theoretical character. From eight to twelve lectures on anatomy, physiology, and the nursing of special cases are delivered in the winter months by one of the assistant honorary surgeons to the senior division, and by the senior house physicians to the juniors. The probationers in their first year also attend classes which are held by me. I deal with nursing duties, the meaning of various surgical and medical terms, catechising a little. There is an examination at the end of each course of lectures and a more or less stiff number of questions have to be answered."

"Can you give me any illustrations?"

"The seniors are asked such questions as 'How could you know that a patient was suffering from internal hæmorrhage after an abdominal operation?' 'What do you mean by the term simple fracture, compound fracture, complicated fracture, comminated fracture?' There are five questions, and in each case the matron gives the last. In the paper I am quoting from I asked, 'If required to prepare a room in a private house for an important operation, how would you proceed?'"

"You might, please, quote the matron's question for the juniors?"

"'What special precaution would you observe in the nursing of cases of paralysis and fractured spine?' Here are two of the other questions to juniors, 'Name the most important vessel the blood would pass through during the passage from the left ventricle of the heart to the right auricle?' 'On what portion of the body are you most likely to get pressure sores due to splint or otherwise?' 'How would you prevent splint sores?' Of course there is an excellent practical training."

### The Practical Training.

"I gather that it is also very varied."

"It is both very varied and very heavy. This is the only general hospital in Newcastle, and most of the important cases from Northumberland and Durham come here. There are many accidents at the large works and the pits, some of the patients being in a pitiable state when they arrive. The utmost nursing care and attention are required. In fact, serious cases are in a larger proportion in this hospital than in any hospital in which I have worked before."

"Is there any difficulty in obtaining probationers?"

"No, I cannot say that there is, but many candidates are physically unfit for the heavy work here. We keep our probationers on as staff nurses or sisters. The fact is that very few nurses trained elsewhere would put up with the heavy work in the wards and the discomforts outside."

### The Question of Pensions.

"Have you anything to say about uniform or recreation?"

"Uniform is not provided by the committee, and the nurses can wear it or not as they please out of doors. We have tennis-courts here, but the recreation they have mainly to find for themselves. There is no library nor supply of magazines or newspapers provided by the committee."

"I have just time to ask one more question. Is the Infirmary affiliated to the Royal National Pension Fund for Nurses?"

"I am sorry to say it is not. The committee have occasionally given pensions to nurses who have done particularly well and served some time, but apparently they cannot see their way to joining the Pension Fund. Perhaps, after we are settled at Leazes Park the position may be more favourable for this."

### Novelties for Nurses.

BY OUR SHOPPING CORRESPONDENT.

### LEMONA TOILET CREAM.

THIS is a delightful new addition to the toilet table. It is most refreshing when applied to the face and hands, which it leaves pleasantly smooth and soft. The manufacturers claim that it is produced solely from the lemon, and being, unlike most toilet creams, a vegetable preparation, it does not increase the growth of hair on the face. In the case of chapped hands it is most healing; it is an antidote to hard water, is cleansing, and a very good protection against sun-burn. Lemona is sold in tubes, and can be purchased at drug stores or from the Lemona Company, 13 Bartlett's Buildings, Holborn Circus, London, E.C.

### NOURISHING CHOCOLATE.

(CADBURY BROS., LIMITED, BOURNEVILLE, BIRMINGHAM.)

WE all know that Cadbury's chocolate now holds a foremost place amongst chocolates, both British and foreign. For purity and nutritive properties it cannot be excelled. For this reason their chocolate represents one of the most useful forms of diet. It is acceptable both to young and old, to people in health and sickness. Now that the holiday season has begun, Cadbury's Milk Chocolate especially will serve as a boon to many travellers. It is impossible to guard against all contingencies, but cautious persons would do well to provide themselves with a supply of milk chocolate tablets, and so be rendered secure from the exhaustion and discomfort of hunger. There is no other article of food, so conveniently carried, that provides in so small a space anything so nutritious, pleasant, and digestible.

### THE BEST EAU DE COLOGNE AND A NEW SCENT.

THE address of the depôt at which these perfumes can be obtained is 62 New Bond Street, W.

### Nursing in Workhouse Infirmaries.

THE DEPARTMENTAL REPORT.

On Monday, by permission of the Hon. Mrs. J. G. Talbot, a conference was held at 10 Great George Street, under the auspices of the Workhouse Infirmary Nursing Association, to discuss the report of the Departmental Committee on Nursing appointed by the President of the Local Government Board.

The chair was taken by the Right Hon. J. G. Talbot, M.P., who, in the course of a few remarks, said that the fact of such a conference was in itself a very encouraging sign, and showed what an enormous stride had been made in the present generation. Thirty or forty years ago, the very idea of obtaining paid nurses for workhouse infirmaries was considered to be most advanced, and they must now take care that the great progress they had made was maintained. The danger they had to ward against was lest an inferior class of nurses should be allowed to creep in.

Miss Wilson, treasurer of the Association, read several letters which had been received regarding the proposed creation of "qualified nurses," all of which were in opposition to this scheme.

Mr. H. Bonham-Carter said that hitherto no nurse was considered qualified unless she had undergone a three years' training in one of the major training schools and had passed a successful examination. The proposed regulations enacted that a probationer, after one year's training in a minor training school, might obtain a formal certificate, be styled a "qualified nurse," and be recognised by the Local Government Board as a proper person to fill the post of workhouse nurse. The duty of a trained nurse was to learn to carry out intelligently the orders of the medical officer respecting the patient; to be able to do this it was essential that she should go through a regular and systematic training in her duties. In most of the discussions regarding the essentials of training for a nurse the moral qualities that were necessary were not sufficiently recognised, *i.e.*, patience, love of order, tact, sympathy, strict obedience, accuracy of observation. It required an experienced and highly-trained superintendent nurse to be able to ascertain whether a nurse possessed these qualities, and in the smaller workhouses the superintendents could not be expected to be quite so high a grade. The foundation of a nurse's training depended upon her moral qualities as well as upon the technical instruction she received. The proposal of the Departmental Committee was that the training and education should be of a lower grade, so that the qualifications of a nurse would also be inferior and that fact would damage the status of nurses generally.

Miss Brodie Hall, as a Poor Law guardian, said that the Union to which she belonged was indebted to the Workhouse Infirmary Nursing Association for the supply of nurses. She would much like to see a central organisation appointed for the supply of nurses by which they could be moved from one union to another. She recommended the reduction in the number of superintendent nurses and the appointment of the matron to act as matron-nurse. She also advocated the claims of the smaller workhouse infirmaries to be considered major training schools, urging that efficiency and a high standard of training should be reckoned in place of the mere number of beds.

Mr. Heywood Johnstone, M.P., said that if the proposal of the Local Government Board came into operation, and a nurse was considered "qualified" after 12 months passed in picking up elementary information, she would have little inducement to remain a Poor Law nurse but would go in for the much better paid branch of private nursing, to the great injury of the properly trained nurses.

Miss Louisa Twining, who was received with much applause, remarked that she had been induced to look up the report of evidence she had given before a Parliamentary Committee 42 years ago, and she found that most of the points she had recommended to the committee of 1903 were the same as those mentioned in 1861. The first and most important improvement in Poor Law reform that she wished to obtain was the appointment of women as inspectors. Women inspectors were now required in factories; how much more then in country workhouses, where the matron was untrained and the medical officer non-resident? Another reform which she wished to see brought about was the amalgamation of smaller workhouse infirmaries. The position of the matrons in the separated infirmaries should be revised and altered so as to be exactly the same as that of a matron in a hospital. The only solution of the difficulties of nursing in the smaller workhouses was the adoption of a scheme of centralisation.

Miss C. J. Wood, speaking of the danger to the public of these so-called "qualified nurses," urged that the nursing profession must be considered as a whole, and that what affected one branch affected all the others. She asked if a Government department would be justified in launching upon the public nurses without the proper amount of training.

Miss Amy Hughes gave a graphic description of her experience as superintendent nurse of an unseparated workhouse infirmary containing 420 beds. She was strongly of opinion that the medical officer and superintendent nurse should be represented on the Board of Guardians.

After some discussion Mr. Talbot moved a resolution to the following effect:—

"That this conference desires to lay before the President of the Local Government Board its judgment on some recommendations contained in the report of the Departmental Committee on nursing of the sick poor in workhouses, and would ask that a deputation be received by the Board."

This was unanimously adopted.

Miss Gibson, matron of Birmingham Infirmary, was unable to attend the conference.

### Royal National Pension Fund for Nurses.

MEETING AT THE WESTERN INFIRMARY, GLASGOW.

On Monday afternoon last a meeting was held in the Conservatory of the Western Infirmary, Glasgow, for the purpose of increasing the interest in the Royal National Pension Fund for Nurses. There was a large and representative attendance, which included the Lord Provost, Sir John Ure Primrose, Bart.; Mr. J. D. Hedderwick, chairman of the Royal Infirmary; Mr. James Boyd, chairman of the House Committee of the Western Infirmary; Dr. Beatson, Dr. Thomas, Royal Infirmary; Dr. Mackintosh, superintendent of the Western Infirmary. The number of nurses present was considerable, and every hospital and institution public and private sent representatives. The Lord Provost presided and introduced Sir Henry Burdett as the founder and deputy chairman of the Fund, who was present in order to explain its aims and objects.

He said:—They all recognised that in their lives' history there was the rainy day which came to everyone, and that any institution which took hostages from the future for the well-being of the individual commended itself to every right-thinking man and woman. Sir Henry then proceeded to explain that the Royal National Pension Fund for Nurses was the greatest provident organisation for women workers in the whole world. It was so constituted and founded as to make nursing a perfectly safe occupation for women. By joining this fund in the earlier years of her engagement, when her contract was very cheap, a nurse might become independent and safe all her life. The basis of the Fund was love—to look after those who gave the best years of their life and health to the care of the sick. It was managed by men who controlled the greatest national forces in the Empire, who gave their time and the best of their knowledge to see that the nurses' money was not only safely but properly invested.

The Pension Fund enabled those who took out a policy to have a definite annual payment on reaching a certain age. For this purpose £80,000 had been invested. Then, again, a sick policy entitled the holder to an allowance which would make her independent, no matter what her circumstances might be. Should the sick allowance not be sufficient, there was a benevolent fund out of which a grant was made according to the merits of the case. If the nurses wished to leave the Fund, they were entitled to what they had paid into it with compound interest at 2½ per cent. Should the withdrawal from the Fund take place very soon after joining, there was a condition that a reduction of 5 per cent. would be made in order that the nurses in the Fund might not have to pay anything out of their savings for the other's want of continuous thrift. The Fund was practically a savings bank for nurses, all of whom were entitled at any time to the amount saved. Their income per annum was £120,000, of which £95,000 represented premiums for nurses. The Fund had been working for 14 years, and the income from investments now exceeded £25,000 a year.

Sir Henry, in concluding his remarks, appealed to the managers of the various nursing institutions in Glasgow to do all they could to further the interests of the Fund among their nurses, and urged the desirability of federating their institutions with the Fund for the purpose of making some provision for their nurses after the age of 50. The efficiency of such institutions rested on something very much more precious than bricks and mortar—viz., the workers who took all the risks represented by a lifelong service for the sick.

### Everybody's Opinion.

### OCCUPATION FOR CHRONIC INVALIDS.

"E. R." writes: Many readers of your paper must be in charge of chronic invalids to whom some occupation of interest would be a great benefit. May I call the attention of such to the Invalids' Auxiliary to the Edinburgh Medical Mission? There is no subscription, but each member is asked to take an interest in the Mission, and to send a piece of work to the annual sale of work held in November. The proceeds of the sale, which last year amounted to £142, are divided among different medical missions, home and foreign. Anyone wishing to join can obtain a report and all particulars from Miss Sym, 21 Belgrave Crescent, Edinburgh. I know from my own experience how much some outside interest lightens the enforced idleness and tedium of ill health.

### Presentations.

BRISTOL ROYAL HOSPITAL FOR SICK CHILDREN AND WOMEN.—Miss Frances L. Peake, sister of the medical floor of the Bristol Royal Hospital for Sick Children, on leaving for the Brook Hospital, London, has been presented with a very handsome silver toilet set by the matron and nursing staff.

LODGE MOOR HOSPITAL, SHEFFIELD. On the occasion of leaving Lodge Moor Hospital to become matron of the Grimsby General Hospital, Miss Frances Crichton, the assistant matron, received the following presents from the sisters and nursing staff:—Oak tray, afternoon Dresden china tea set, and mother-of-pearl sweet dish, as a token of their good wishes.

### Appointments.

ADDENBROOKE'S HOSPITAL, CAMBRIDGE.—Miss Alice Harris has been appointed staff nurse. She was trained at Middlesex Hospital, London, where she has since been staff nurse. She holds the L.O.S. certificate.

BEXLEY COTTAGE HOSPITAL.—Miss Helena McKinley has been appointed nurse matron. She was trained at Guy's Hospital, London, and her last appointment was that of night sister at the Hospital for Diseases of the Throat, Golden Square, London.

BURTON-ON-TRENT GENERAL INFIRMARY.—Miss Florence Therburn and Miss Mary Savage have been appointed sisters. Miss Therburn was trained at Derbyshire Royal Infirmary. Miss Savage was trained at Bolton Infirmary, and has since been sister at the Park Hospital, Hither Green, and attached to the Blackheath Nursing Institute.

COTTAGE HOSPITAL, EAST COWES.—Miss Florence H. Bailey has been appointed staff nurse. She was trained at Bethnal Green Infirmary, London.

EPSOM COTTAGE HOSPITAL.—Miss Susie Dunham has been appointed matron and Miss L. J. Rodgers staff nurse. Miss Dunham was trained at the London Hospital, and remained as staff nurse for seven years. She has also been matron of Bexley Cottage Hospital for two years. Miss Rodgers was trained at Camberwell Infirmary, and has since been sister at Portsmouth Union Infirmary.

LODGE MOOR HOSPITAL, SHEFFIELD.—Miss Frances Pritchard has been appointed night superintendent. She was trained at Fir Vale Infirmary, Sheffield, and has since been assistant nurse and charge nurse in the same institution. She has also been attached to the Brooklyn Private Nursing Institute, Anerley, London, and was for a short time charge nurse at the South Eastern Hospital, London.

MANCHESTER HOSPITAL FOR CONSUMPTION, BOWDEN.—Miss F. E. Gardiner has been appointed matron. She was trained at Derby Infirmary, and has since been matron of the Birmingham and Midland Counties Sanatorium for nine years, and matron of Nordrach-on-Dee Sanatorium for fourteen months.

MARGATE COTTAGE HOSPITAL.—Miss Florence Winch has been appointed staff nurse. She was trained at St. Saviour's Infirmary, East Dulwich, and has since been nurse at Darenth Schools for Imbecile Children, and at North Staffordshire Infirmary.

MARTHEY WORKHOUSE INFIRMARY, NEAR WORCESTER.—Miss Mary Sharp has been appointed superintendent nurse. She was trained at Chorlton Union Infirmary, and has since been midwifery charge nurse at King's Norton Union.

POPLAR AND STEPNEY SICK ASYLUM, BROMLEY.—Miss Florence A. Foyster has been appointed assistant matron. She was trained at Whitechapel Infirmary, where she has since been for the last three and a half years night superintendent. She holds the L.O.S. certificate.

ST. LEONARD'S INFIRMARY, SHOREDITCH.—Miss Fanny Estelle Griffiths has been appointed staff nurse. She was trained at Croydon Union Infirmary, and has since been staff nurse at the City of London Hospital, the Park Fever Hospital, and St. Giles's Workhouse, Endell Street, London. She has also been employed by the Colonial Nursing Association.

STEYNING UNION INFIRMARY, SHOREHAM.—Miss Grace Ward has been appointed assistant superintendent and charge nurse. She was trained at the Camberwell Infirmary, and has since been staff nurse for two years at the Gordon Hospital, Vauxhall Bridge Road, London.

YORK CORPORATION FEVER HOSPITAL.—Miss L. Bell has been appointed charge nurse. She was trained at the City Hospital, Bradford, and has since been assistant nurse at the City Hospital, Beckett Street, Leeds, and charge nurse at the Isolation Hospital, Crewe.

### Echoes from the Outside World.

### The King's Birthday.

FRIDAY was the King's birthday, and in all parts of the country there were rejoicings. In London the popular event of the day was the ceremony of trooping the colour in the Horse Guards Parade, which was witnessed by thousands of spectators, including the Khedive of Egypt, in lovely weather. The windows of the public buildings overlooking the parade ground were brightened by the ladies' gay frocks and by a trimming of coloured bunting, those actually behind the base being reserved for the Queen, the Princess of Wales, and other members of the Royal party. The King and the Prince of Wales, both on horseback, arrived on the stroke of eleven, accompanied by a brilliantly-caparisoned staff, with the Commander-in-Chief at their head. The approach of his Majesty was heralded by the playing of the National Anthem. The ceremony was, of course, the same as usual, but of unusual interest, it being the first which the King has witnessed since his accession. After the King had inspected the troops, there was a march past of the regiments—Scots Guards, Irish Guards, Grenadiers, and Coldstreams, in all 1,900 strong. At the conclusion of the function, which lasted about an hour, the King, the Queen, and all the members of the Royal Family were loudly cheered by the public as they left the Horse Guards. There were the customary State dinner parties at night, and the West End was illuminated. One of the most notable salutes in the provinces was at Plymouth, where more than 5,000 troops, with the Naval Brigade, lined the shores of the Sound, forming a continuous line ten miles in length, and at noon fired a *feu de joie.* The Royal salutes were fired by the citadel, and the ships in the harbour were dressed rainbow fashion. Vast crowds of spectators witnessed the display.

### Birthday Honours.

ON the occasion of the celebration of his Majesty's birthday the King conferred a large number of honours, including four new peerages, six baronetcies, and nineteen knighthoods. One of the new peers is Sir Edward Lawson, principal proprietor of the *Daily Telegraph*, and another Mr. Watson-Armstrong, nephew and heir of Lord Armstrong, to whose munificence the people of Newcastle-upon-Tyne are largely indebted for the means to build a new infirmary. The new knights include three well-known members of the medical profession, namely Mr. Alfred Fripp, surgeon in ordinary to the King; Dr. E. C. Perry, the popular superintendent at Guy's Hospital; and Dr. Stephen Mackenzie, of the London Hospital. The King has also granted the Imperial Service Medal to several hundred subordinate members of the Civil Service on their retirement after 25 years or more of meritorious service. The recipients include rural and town postmen, prison warders, postal telegraphists, dockyard employés, and messengers.

### The Queen at Regent's Park.

ON Saturday the Queen visited the dog show at Regent's Park under the auspices of the Ladies' Kennel Association. In order to prove her interest in the Association, her Majesty sent a number of dogs to the show, two of whom won prizes, and contributed £100 to its funds. During her stay in the Royal Botanic Gardens, which were looking their best, she was presented with a bouquet of damask and pink roses by the St. Bernard dog Leo, who is the hospital dog at the Harrow Hospital. After a general review of the animals, the Queen witnessed a parade of the prize-winners, and afterwards took tea in the club-room. On the previous day the Duchess of Connaught and her daughters had paid a surprise visit to the exhibition.

### Soldiers and Sailors' Families Association.

PRINCESS LOUISE presided on Friday at the Annual Meeting of the Soldiers and Sailors' Families Association, of which the King is patron and the Queen president. The report was read by Sir James Gildea, and among the interesting points mentioned was the fact that in the last three years and three months the public had entrusted to a voluntary and almost untried organisation upwards of a million and a quarter sterling. Another feature was that more than 1,300 ladies and gentlemen had devoted time, labour, tact, and skill to carry on the work, complicated as it was by many technical questions affecting official allowances; and a third, the harmonious working of the local representatives of the Association with those of local independent funds, resulting at the close of the war in most of the committees of the latter joining the Association, or handing over their unexpected balances. Reference was made to the contribution by the Queen of £5,000 from her War Fund, and it was stated that last year there were 20 candidates for the Homes of Officers' Widows and Daughters, established in 1899, which number had now been increased by 30 more, making altogether 50 ladies awaiting election. Admiral Lord Charles Scott, who was one of the speakers, testified that the recipients of aid had behaved wonderfully well, and Mr. Hayes Fisher, M.P., dwelt on the importance of continuing the work of the organisation now that the war was over. On Wednesday an exhibition and sale in aid of the Fund was opened at Queen's Hall by the Duchess of Albany.

### An Imperial Technical College.

ON Monday Lord Rosebery announced that Messrs. Wernher, Beit and Co., Sir Ernest Cassel, Lord Strathcona, and other public-spirited citizens of the metropolis have offered to contribute about £300,000 for the creation of a scientific university in London, of the same class as the famous College of Applied Sciences at Charlottenburg in Germany, on condition that the London County Council undertake the upkeep, which it is estimated will cost at the outset about £20,000 a year. The Exhibition Commissioners have offered a site of four acres at South Kensington, and the sum to provide the buildings is to be administered by a trust, consisting of Lord Rosebery, who will act as chairman, Mr. Balfour, the Duke of Devonshire, Sir Francis Mowatt, Mr. Julius Wernher, Mr. R. B. Haldane, the Vice-Chancellor and the Principal of the University of London. If this scheme is carried out, there will be no need for able young men in this country, anxious to equip themselves with the most perfect technical training, to resort to foreign universities or the United States.

### Railway Disaster in Spain

AN appalling railway accident took place on Saturday evening near Cenicero, in Spain. The mail train drawn by two engines was proceeding from Bilbao to Saragossa. The first engine passed safely over the bridge across the river Najerilla, which flows into the Ebro, but the second jumped the rails, broke away from the leading locomotive, and plunged into the river, 30 feet below, dragging the whole of the 18 coaches behind it. Of the passengers it is officially stated that more than a hundred were killed, and a large number injured. The train contained a number of invalids who were going to the sulphur-baths at Fitero, a party of wealthy mineowners of Bilbao, gendarmes on their way to Barcelona to assist in maintaining order among the strikers, and Sisters of Charity. Some of the survivors have lost the power of speech in consequence of nervous shock. A railway guard has been arrested for robbing the body of a dead passenger.

### For Reading to the Sick.

EVENING HYMN.

THE shadows of the evening hours
Fall from the darkening sky;
Upon the fragrance of the flowers
The dews of evening lie:
Before Thy throne, O Lord of Heaven,
We kneel at close of day;
Look on Thy children from on high,
And hear us while we pray.

The sorrows of Thy servants, Lord,
Oh, do not thou despise;
But let the incense of our prayers
Before Thy mercy rise;
The brightness of the coming night
Upon the darkness rolls:
With hopes of future glory chase
The shadows on our souls.

Let peace, Oh Lord, Thy peace, Oh God,
Upon our souls descend;
From midnight fears and perils, Thou
Our trembling hearts defend;
Give us a respite from our toil,
Calm and subdue our woes;
Through the long day we suffer, Lord,
Oh, give us now repose!

*A. Proctor.*

"They drew nigh to the town whither they were going, and He made as though He would go further. But they said: Stay with us, because it is towards evening, and the day is now far spent. And He went in with them."

There is a lesson for us all in this exquisite scene. Our Lord met you on the road of life when you were quite young, and little by little He unfolded to your mind what He had done for you, and asked you to be one of His disciples. And since that day, from time to time, as surely as night follows day, it has seemed—and all through life it will be the same—as though our Lord left you and withdrew Himself from you, especially in times of pain or of disappointment. Then you cry out, and rightly, "Stay with me, for the day is far spent; Lord, stay with me and help me." That prayer is always heard. It is our Lord's way "to make as though He would go further," to lead you on to higher things and make you cling to Him the more by seeming to withdraw Himself; but He is close to you all the time. "He will not leave you." And you can hear His voice, though you cannot see His face or tell what He is doing or going to do for you.

"Stay with me dear, Lord. In Thine agony, Thou hadst no one to watch with Thee; but in my pain and desolation Thou art ever at my side, my constant, unfailing friend; let me realise this mark of Thy love."

*From "A Hundred Readings."*

God, Who through life hath fed you to this day,
Defend you to the end;
His living Bread be still your Staff and Stay;
His Angel still your Friend;
Till daylight fades, and hues of evening fall,
Till shadows cease, and God is All in All.

*R. E. J. A.*

### Notes and Queries.

FOR REGULATIONS SEE PAGE 136.

*Books.*

(125) Will you kindly tell me which you think the best book to study for one soon to train for monthly nursing?—*Inquirer.*

If you are a trained nurse Cullingworth's "Short Manual for Monthly Nurses," price 1s. 6d., will serve as an easy introduction, followed by Haultain's "Practical Midwifery," price 6s. If you are untrained, Jardine's "Text-book of Midwifery," price 6s. (which contains some physiology), and "The Nurses' Dictionary of Medical Terms," by Honnor Morten, will prove useful. The Scientific Press, 28 Southampton Street, Strand, or any bookseller, will get these books for you.

Will you kindly tell me if there is any inexpensive, illustrated book from which an intending probationer could learn the names of the surgical instruments in common use?—*Nitric.*

"Surgical Ward Work," by Miles, price 3s. 6d. net, contains illustrations and descriptions of all the instruments in general use. You can procure it from the Scientific Press, 28 Southampton Street, Strand, or any bookseller.

I shall be indebted to you if you will kindly recommend me a reliable medical dictionary, those which I have in use (including Honnor Morten's) having proved inadequate.—*Inquirer.*

"Holbyn's Dictionary," price 10s. 6d., is excellent and full. The Scientific Press will procure for you any book you require.

*Open-air Treatment.*

(126) Will you kindly tell me the best open-air treatment for a child of 14, some way gone in phthisis. Is the one at Margate suitable, as she is of the working class?—*P.*

It is impossible to advise. The case is one for a doctor.

*Address.*

(127) Some time ago I saw advertised in The Hospital work offered to nurses who wish to improve their income, and as I am not getting wages while training for a year, I should be glad to earn something in my off-duty time. Can you insert address of the advertiser?—*Anxious.*

We cannot give you the address, and hope that you will employ your leisure time in necessary rest and recreation.

*Paris.*

(128) Can you tell me the address of the Ophthalmic Hospital in Paris? I should be very glad if you could tell me how a trained English nurse could obtain work there.—*S. S. S.*

There is no Ophthalmic Hospital in Paris. The matron of the Hertford British Hospital, Rue de Villiers-Levallois-Perret might give you information as to the prospects of English nurses in Paris if you write and enclose stamp for reply.

*Baby Food.*

(129) 1. Will you kindly tell me where I could get rusks for babies? 2. Is it sufficient to pour boiling milk over the rusks, or ought they to be boiled in it? The child is eight months old, weighs 17 lbs., and appears tired of milk. 3. Is it too young for rusks?—*A. M. H.*

1. You can obtain rusks or Robbs' biscuits at any good bakers. 2. Follow directions given on the bags. 3. You should obtain advice of an experienced person as to a change of diet.

*Medico-Psychological Certificate.*

(130) 1. Will you kindly tell me how I can obtain the certificate of the Medico-Psychological Society? I am a trained nurse. (2) Is there any book written on the "Finsen Light Apparatus?"

1. Write to the Registrar, the Medico-Psychological Association, 11 Chandos Street, W. 2. Articles on the subject appeared in The Hospital, April 25th, May 30th, June 6th.

*Testimonials.*

(131) Is it customary to give testimonials to nurses upon their leaving hospitals, private nursing staffs, etc.? So many advertisements state that applicants must produce at least three testimonials. To whom should I apply for them?—*H. E.*

You should apply to the matron, medical staff, and other authorities in hospitals, and to the medical men and their patients for whom you have worked in private nursing.

**Standard Nursing Manuals.**

"The Nursing Profession: How and Where to Train." 2s. net; 2s. 4d. post free.
"Nursing: Its Theory and Practice." (Revised Edition). 3s. 6d. post free.
"Surgical Ward Work and Nursing." (Revised Edition). 3s. 6d. net.; 3s. 10d. post free.
"Practical Handbook of Midwifery." (New Edition). 6s. net; 6s. 3d. post free.
"Notes on Pharmacy and Dispensing for Nurses." 1s. post free.
"Fevers and Infectious Diseases." 1s. post free.
"The Art of Massage." (New Edition). 6s. post free.

